# Lipid Raft, Regulator of Plasmodesmal Callose Homeostasis

**DOI:** 10.3390/plants6020015

**Published:** 2017-04-03

**Authors:** Arya Bagus Boedi Iswanto, Jae-Yean Kim

**Affiliations:** Division of Applied Life Science (BK21 Plus program), Plant Molecular Biology and Biotechnology Research Center, Gyeongsang National University, Jinju 660-701, Korea; aryabagus62@gmail.com

**Keywords:** lipid raft, sterol, sphingolipid, plasmodesmata, plasmodesmata callose

## Abstract

The specialized plasma membrane microdomains known as lipid rafts are enriched by sterols and sphingolipids. Lipid rafts facilitate cellular signal transduction by controlling the assembly of signaling molecules and membrane protein trafficking. Another specialized compartment of plant cells, the plasmodesmata (PD), which regulates the symplasmic intercellular movement of certain molecules between adjacent cells, also contains a phospholipid bilayer membrane. The dynamic permeability of plasmodesmata (PDs) is highly controlled by plasmodesmata callose (PDC), which is synthesized by CALLOSE SYNTHASES (CalS) and degraded by β-1,3-GLUCANASES (BGs). In recent studies, remarkable observations regarding the correlation between lipid raft formation and symplasmic intracellular trafficking have been reported, and the PDC has been suggested to be the regulator of the size exclusion limit of PDs. It has been suggested that the alteration of lipid raft substances impairs PDC homeostasis, subsequently affecting PD functions. In this review, we discuss the substantial role of membrane lipid rafts in PDC homeostasis and provide avenues for understanding the fundamental behavior of the lipid raft–processed PDC.

## 1. Lipid Raft Components

The plasma membrane is a biological compartment shielding the contents and substances of the entire cell. The plasma membrane allows the formation of a complex intracellular organization, enabling cellular activities to be substantially regulated. Therefore, its unique structure plays a critical role in biological processes including the transduction of various signals and serves as a selectively permeable barrier to prevent the unrestricted exchange of molecules from one side to the other [1]. Furthermore, this compartment, which encircles the cell, mediates intercellular interactions for the exchange of materials and information between cells [2].

**Figure 1:**
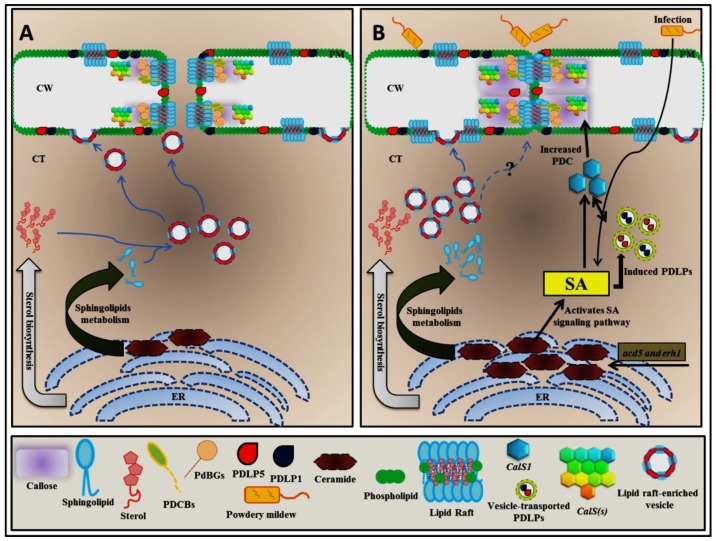
Hypothetical model of how ceramides could control PD permeability. Sterol and sphingolipid biosynthesis begins at the endoplasmic reticulum (ER), and these molecules are subsequently transported to the plasma membrane by the vesicle-mediated exocytosis pathway to form lipid rafts, preferentially at plasmodesma (PD) membranes. Eventually, PD membranes are enriched by lipid raft formation (**A**). The excessive ceramide in *acd5* and *erh1* mutant plants enables the salicylic acid (SA)-mediated upregulation of *PD LOCATED PROTEIN* (*PDLP*) and *CALLOSE SYNTHASE1* (*CalS1*) transcript levels to induce plasmodesmata callose (PDC) accumulation (**B**); SA-mediated PDLP and CalS1 activation can also be directly upregulated during infection with a biotrophic pathogen such as powdery mildew (**B**). Blue arrows, trafficking; black arrows, signaling; Question mark, not enough evidence that explains how the lipid raft–enriched vesicle controls plasmodesmata callose directly.

**Figure 2:**
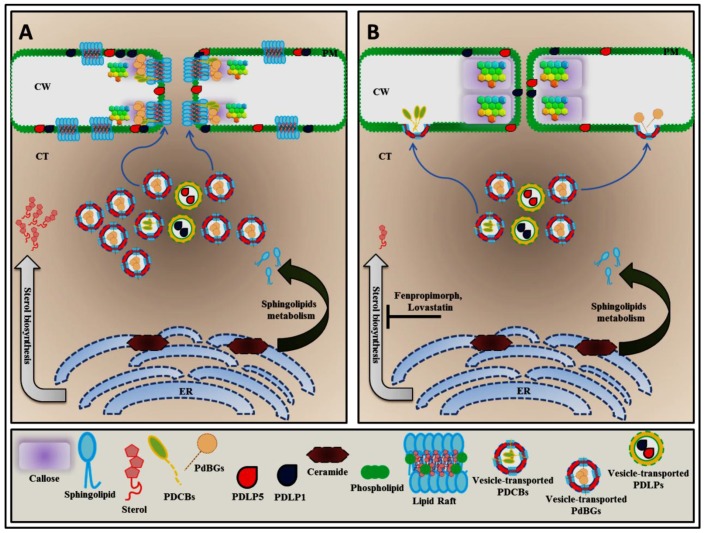
Localization of GPI-anchored plasmodesmata (PD) proteins are controlled by lipid rafts. GPI-anchored PD proteins such as plasmodesmata callose binding (PDCB) proteins and plasmodesmal-localized β-1,3-glucanases (PDBGs) are synthesized in the endoplasmic reticulum (ER). These two proteins may require lipid raft–enriched vesicle-mediated exocytosis machinery to reach both the PD plasma membrane and the cellular plasma membrane as their target locations (**A**). An excessive sterol amount is able to induce the lipid raft–enriched vesicle-mediated exocytosis of PDCBs and PDBGs to regulate symplastic nanochannels by governing plasmodesmata callose (PDC) accumulation (**A**). The disruption of the sterol biosynthesis pathway with fenpropimorph or lovastatin affects the transport system of GPI-anchored PD proteins, preventing the proper localization of these two proteins (**B**).

A major feature of the plasma membrane is the lipid bilayer, whose function in living organisms has been determined in detail, allowing for the emergence of fundamental concepts concerning the function of the cellular membrane [3,4]. Some domains of the plasma membrane phospholipid bilayer are enriched in specific lipids that reside in the plane of the membrane, and these domains are termed lipid rafts (Figure 1A and Figure 2A) [3,5,6]. These specialized domains are defined as dynamic and small (10 to 200 nm) plasma membrane domains that are enriched in sterol and typical phytosphingolipids, such as glycosylinositolphosphoceramides, and that contain a low amount of unsaturated phospholipids [5,7,8,9,10,11]. These substances are packed together to form a highly ordered structure distinct from the surrounding disordered area to induce lateral heterogeneity, enabling important biological functions related to membrane signaling and the stabilization of protein-protein, protein-lipid and lipid-lipid interactions [7,8,10]. The presence of lipid rafts in plants was initially identified by analyzing a low-density, Triton X-100–insoluble fraction isolated from tobacco (*Nicotiana tabacum*), which exhibited a protein composition obviously different from that of the general plasma membrane (PM), with an excess of signaling proteins such as heterotrimeric G-proteins and glycosylphosphatidylinositol (GPI)-anchored proteins [12,13,14]. In addition, lipid raft analyses have also been conducted in *Arabidopsis thaliana* along with the characterization of particular lipid compositions from detergent-insoluble membranes (DIMs), providing a new understanding of plant lipid rafts [14,15].

Some membrane proteins, such as glycosylphosphatidylinositol-anchored proteins and acylated cytosolic proteins, show a preferential association with lipid rafts, thereby facilitating various biological functions and dynamic processes, including membrane trafficking, protein sorting, cell polarity and signal transduction [16,17,18]. The plasma membrane has exofacial and cytofacial leaflets, which cause the phospholipid bilayer to differ in electrical charge, fluidity and the activation of certain proteins. Exofacial proteins anchored by glycophosphatidylinositol (GPI) anchors preferentially localize into lipid rafts, whereas cytofacial proteins are modified by saturated fatty acids such as palmitoyl or myristoyl groups [17,19,20,21,22]. Fluorescence microscopy has allowed for the observation of phase separation in lipid membranes [23], and fluorescently labeled lipids or lipophilic dyes have been commonly used as imaging agents [24].

As mentioned above, plant lipid rafts possess an enriched sterol and phytosphingolipid molecules, and therefore the action of plant lipid rafts is highly influenced by sterol and sphingolipid biosynthesis, as the production of these molecules affects plant lipid raft organization and behavior (Figure 1A). In mammalian cell experiments, cholesterol has been suggested to be an important lipid raft component and has been shown to play a critical role in lipid raft stability and organization [25]. In particular, the depletion or perturbation of cell membrane–associated cholesterol diminishes the functions of intact lipid raft–associated membrane components [18,25]. Sterol molecules vary in prokaryotic and eukaryotic cells [26]. In *Arabidopsis thaliana*, the most abundant sterols are cholesterol, sitosterol, stigmasterol and campesterol, and among these, sitosterol is the most abundant phytosterol [27]. Sphingolipids are described as ubiquitous components of cellular membranes and are composed of a long-chain sphingoid base with one amide-linked fatty acyl chain and a polar head group, and sphingosine is the most prominent long-chain sphingoid base [28,29]. Sphingomyelin is the major phosphosphingolipid found in animal cells; however, it is not detected in plant cells. Intriguingly, glycosylinositolphosphorylceramides (GIPCs) are the most abundant in plant cells, but these have disappeared in animal cells [29].

## 2. The Action of Plasmodesmata Callose

Plasmodesmata (PDs) are dynamic symplasmic nanochannels that are localized in the plant cell wall and connect the cytoplasm spaces and endoplasmic reticulum compartments of adjacent cells [30,31,32,33,34]. PDs mediate the symplasmic movement of small molecules such as water, ions, small nucleotides, phytohormones and other solutes (amino acids and sugar). Relatively larger molecules, including peptides and small proteins, are also able to be symplasmically moved through PDs [34,35]. In addition, it has been reported that PDs facilitate the cell-to-cell trafficking of homeodomain transcription factors (TFs) and other proteins through an actively regulated process [31,36,37]. Indeed, the active cell-to-cell communication machinery presumably involves protein-protein interactions at PDs to regulate the size exclusion limit (SEL). The PD SEL can be described as the size of the largest molecules that are able to pass through the PD [38]. The size of the PD SEL can be controlled by callose deposition in the neck region of the PD aperture [39,40,41]; therefore, a lack of plasmodesmata callose (PDC) presumably enhances the movement of molecules through PD channels [42].

Callose is a polysaccharide that is produced by callose synthase and degraded by β-1,3-glucanases. Callose is widely found in higher plants, in which it is a component of specialized cell walls or cell wall–associated structures at particular stages of growth and differentiation. Callose plays diverse roles during plant growth and development in order to ensure proper growth. In addition, it is particularly involved in the plant responses to both biotic and abiotic environmental stresses [35,43,44,45]. Furthermore, biochemical and biological studies of callose have shown that callose plays important roles in plant developmental processes, including cell division, organogenesis, microsporogenesis, pollen germination and tube growth, fertilization, embryogenesis, fruit ripening, seed germination, mobilization of storage reserves in the endosperm of cereal grains, bud dormancy, and responses to wounding, cold, ozone and the other stresses [46,47,48,49,50,51,52,53,54,55].

Twelve *GLUCAN SYNTHASE-LIKE* (*GSL*) genes (also known as *CALLOSE SYNTHASE* (*CalS*)) have been identified and characterized in *Arabidopsis thaliana*. Among the 12 callose synthases identified in *Arabidopsis*, five (*CalS10/GSL8*, *CalS7/GSL7*, *CalS3/GSL12*, *CalS1/GSL6* and *CalS8/GSL4*) have been found to play a direct role in PDC deposition. Correspondingly, previous studies have suggested that *CalS10/GSL8* strongly regulates PDC deposition to maintain cell-to-cell permeability in plants [56,57,58], and *gsl8* homozygote mutants are seedling-lethal [46,59]. Another callose synthase gene, *CalS7/GSL7*, is responsible for PDC in the phloem of vascular tissue. The observation of callose deposition at sieve elements in the *cals7* mutant reveals that the reduction of PDC deposition during sieve element development and maturation results in a disordered sieve element pattern [58]. Recent studies on the regulation of PDs have also implicated the *CalS3/GSL12* gene; *CalS3* participates in PDC formation specifically at the stem cell niche and stele [40]. Alterations in vascular patterning are caused by two allelic gain-of-function mutations, *cals3-1d* and *cals3-2d*. The overproduction of PDC in the *cals3-d* mutant impairs molecular trafficking through PDs, especially during root development [60]. Moreover, the disruption of cell-to-cell communication at PDs has also been shown by *icals3m*, an inducible vector that enables the overexpression of *cals3*. Loss of symplasmic signaling in the activation of *icals3m* expression significantly affects root development and the gravitropism response [61]. Since PDC production is involved in the response to some environmental stresses, callose synthase genes controlled by environmental stresses were screened and identified. Two genes, *CalS1/GSL6* and *CalS8/GSL4*, have different roles in responding to certain stresses. *CalS1* is necessary to modulate PDC deposition during pathogen infection by activating the salicylic acid pathway, whereas *CalS8* is tightly associated with the reactive oxygen species (ROS) produced during wounding to control PDC deposition [62].

Polar auxin transport (PAT) is an active process that controls the distribution of the hormone auxin in plants. PAT is an essential process for forming and maintaining the auxin gradient during the phototropic response. However, effective auxin gradient formation also requires a tight cooperation of PDs. Remarkably, excessive PDC accumulation promotes asymmetric auxin distribution in plants, and eventually the side that has more auxin elongates faster than the other side [56,63,64,65]. A recent study has shown that the asymmetric distribution of auxin during tropism is perturbed in *dsGSL8* RNAi plants, and the seedlings are eventually defective in either phototropism or gravitropism, mainly due to PDC depletion [56].

## 3. Sphingolipid and Sterol Biosynthesis Pathway Involved in Plasmodesmata Callose Maintenance

As described above, sphingolipids along with sterols are enriched in detergent-resistant membranes termed lipid rafts and have been linked to certain biological processes and cellular activities, including the sorting and trafficking of specific plasma membrane proteins, and possibly function as signaling molecules to initiate programmed cell death in plants. Moreover, phytosphingosine-1-phosphate has been identified to mediate abscisic acid–dependent guard cell closure by transduction through the unique prototypical G-protein α-subunit GPA1 [28]. Recently, the disruption of a sphingolipid ceramide kinase gene has also been characterized as the basis for the enhanced rate of apoptosis in the *Arabidopsis thaliana accelerated cell death5* (*acd5*) mutant, suggesting that levels of ceramides in sphingolipids control programmed cell death in plants. Correspondingly, in response to *Botrytis cinerea* infection, an *acd5* mutant exhibited more severe disease symptoms, smaller papillae, decreased callose deposition and increased apoplastic and mitochondrial ROS compared to the wild-type plants [66]. Moreover, excessive ceramide accumulation in an *acer-1* mutant resulted in increased plant susceptibility to *Pseudomonas syringae* and more sensitivity to salt stress than the wild-type plant [67]. *Arabidopsis* possesses three genes encoding ceramide synthases with distinct substrate specificities; the *LONGEVITY ASSURANCE GENE ONE HOMOLOG1* (*LOH1*; At3g25540) and *LOH3* (At1g19260)-encoded ceramide synthases use very-long-chain fatty acyl-CoA and trihydroxy long-chain base (LCB) substrates, and the *LOH2* (At3g19260)-encoded ceramide synthase uses palmitoyl-CoA and dihydroxy LCB substrates [68]. However, *LOH2* overexpression resulted in an excessive amount of sphingolipids with C16 fatty acid and dihydroxy LCB ceramides, followed by programmed cell death symptoms and induced salicylic acid (SA) accumulation [69]. In contrast, the *enhancing rpw8-mediated hypersensitive response-like cell death 1* (*erh1*) mutant, exhibiting high ceramide accumulation and the loss of inositol phosphorylceramide synthase (IPCS) activity, showed enhanced RPW8-mediated hypersensitive response-like cell death and resistance to the biotrophic pathogen powdery mildew, which is associated with ceramide accumulation and possibly involves PDC accumulation (Figure 1B). Correspondingly, in the *acd5* mutant, ceramide accumulation also induces programmed cell death and resistance against powdery mildew infection. It seems likely that the excessive ceramide amount in the *acd5* and *erh1* mutants confers resistance against biotrophic pathogens (powdery mildew) rather than hemibiotrophic pathogens such as *Botrytis cinerea.* and *P. syringae.* (Figure 1B) [70].

Since PD permeability is dynamically regulated by PDC, several studies have been conducted to identify proteins that influence PDC turnover and its stabilization at the PD aperture. Furthermore, lipidomic analyses of purified PDs have revealed that PD membr anes contain phospholipids with an excessive amount of saturated lipids compared to the plasma membrane and that GIPCs are predominantly found in PD membranes [6,71]. Endogenous alteration of sterol components can be accomplished by nutritional manipulations in sterol auxotrophic species, such as plants. On the other hand, drug treatment can also be applied to alter sterol biosynthesis profiles. There are also sterol synthesis inhibitors for various steps of sterol biosynthesis (Figure 2B). Microdomain lipid profiling has indicated that sterol biosynthesis inhibitors affect PDC homeostasis. In *Arabidopsis thaliana*, the presence of fenpropimorph, which inhibits fecosterol-to-episterol conversion [72], resulted in increased PDC accumulation after 24 h of treatment. Intriguingly, two GPI-anchored plasmodesmata proteins (GPI-APPs), PDCB1 and PDBG2, were also mislocalized in response to fenpropimorph and lovastatin (Figure 2B) [6].

## 4. Alteration of Sphingolipid Homeostasis Controls *PDLP5 (PLASMODESMATA-LOCATED PROTEIN 5)* Expression through Salicylic Acid (SA)-Dependent Pathway

PD-associated proteins have been experimentally described to participate in PD function, thus it is not surprising that proteins involved in PDC turnover also localize to PDs and are often involved in PD regulation. In addition, several studies have implicated many different cellular processes in the alteration of ceramide and sphingolipid metabolisms, and these processes are associated with plant defense pathways [66,67,73] and include salicylic acid (SA) machinery [70] as well as sphingolipid homeostasis [69,74] . For example, a cell death phenotype in *acd5* and *erh1* is due to an SA-dependent pathway. Abolishing the *ACD5* and *ERH1* gene functions results in disproportionate ceramide to ceramide-1-phosphate and ceramide to inositol phosphorylceramide (IPC) ratios, respectively, causing a high ceramide concentration [66,70]. Subsequently, this high ceramide concentration initiates SA-mediated programmed cell death by upregulating *PDLP5* to protect against biotrophic pathogens (Figure 1B). Previous studies have shown that PLASMODESMATA-LOCATED PROTEINS (PDLPs) are partially associated with PD channels [75,76,77,78]. In *Arabidopsis thaliana*, there are eight members of the PDLP family, and these members contain two extracellular DUF26 domains in the N-terminus, accompanied by a transmembrane domain (TMD) and short cytoplasmic tail in the C-terminus [78]. PDLP5 acts as the molecular link between PD function and the initiation of SA-induced programmed cell death. It has been demonstrated that *PDLP5* is upregulated in response to SA and that PDLP5 also controls PDC deposition to close PDs in response to SA [76]. Subsequently, the loss of PDLP5 activity results in an enhanced PD permeability phenotype and increased susceptibility to bacterial infection [79]. Moreover, a recent study on how SA-mediated *PDLP5* activation regulates the plant immune system showed that *CalS1* appears to be a key component in SA-dependent PD regulation. In the presence of SA, the transcript levels of *PDLP5* and *CalS1* are highly upregulated, whereas the transcript levels of the other *CalS* genes are not significantly enhanced. SA-mediated *PDLP5* activation is required to close PDs by regulating PDC during pathogen infection, and the *cals1-1* mutant failed to increase PDC accumulation or change PD permeability when this plant was treated with SA and *P. syringae.* This result indicates that *CalS1* and *PDLP5* are strongly associated with PD regulation to control PDC accumulation during SA-mediated immune responses (Figure 1B) [62].

## 5. Plasmodesmal Localization of GPI-Anchored Plasmodesmata Proteins is Regulated by Lipid Rafts

Another specific PD-associated protein, PLASMODESMATA CALLOSE BINDING1 (PDCB1), contains a CBM43 functional domain that facilitates callose binding activity, and this protein is therefore located at sites of callose deposition. Recently, PDCB1 was shown to cosegregate with the sphingolipid- and sterol-rich microdomains [6]. Based on a structural domain analysis, PDCB1 protein contains a X8 domain that is responsible for its callose binding activity and the glycophosphatidylinositol (GPI) anchor sequence at the C-terminus, so it should be noted that this protein is preferentially localized in lipid rafts (Figure 2A) [71,80]. On the other hand, one class of cellular factors that is responsible for callose turnover is the 1,3-β-d-glucanases (BGs), which contain a GH17 domain to specifically recognize callose. Among 50 BGs that have been characterized in *Arabidopsis*, some members, including AtBG_papp and PDBG2, possess a predicted C-terminal GPI-anchor attachment motif for targeting to the membrane. During targeting and posttranslational modification, the GPI anchor attachment site is cleaved, and the mature GPI is attached, which is necessary for target localization [6,39]. Recently, using a comparative analysis of PD targeting of the PD and non-PD GPI-anchored proteins, Zavaliev et al. (2016) showed that that GPI modification is necessary and sufficient for PD targeting of both AtBG_papp and PDCB1 [81]. As mentioned above, sterol biosynthesis disruption affects the targeting of the GPI-anchored proteins PDCB1 and PDBG2 to primary PDs and their modulation of PDC accumulation (Figure 2B), due to defects in lipid raft formation [6].

## 6. Conclusions

The investigations of lipid rafts have involved diverse biological concepts, especially in plant cells. This unique membrane has been described in detail by research groups to provide an understanding of the roles of plant lipid rafts. Recent study on plant lipid rafts has focused on plasmodesmata callose (PDC) accumulation in the control of symplasmic channels [6]. Lipid raft–modulated PDC accumulation is dependent on sterol and sphingolipid homeostasis, as these two components are used to form lipid rafts. Furthermore, sterol depletion results in the mislocalization of two specific GPI-anchored PD proteins, PDCBs and PDBGs, which regulate PDC deposition and degrade PDC, respectively. The mislocalization of these two GPI-anchored PD proteins results in an excessive amount of PDC deposition. Additionally, disruption in lipid rafts can affect the targeting of other lipid raft–enriched proteins such as Grain setting defect1 (GSD1), Remorins (REMs) and StRemorin1.3 which modulates PDC levels [82,83,84]. It was shown that GSD1 regulates PD conductance by interacting with ACTIN in association with the PDCB [84]. In addition, the existence of sphingolipids in the lipid raft is also required to maintain the equilibrium of certain signaling machineries in plant cell systems. A direct link between the localization of PDCBs and PDBGs and sphingolipids has not yet been elucidated. However, recent study concerning sphingolipid function in the callose turnover has demonstrated the involvement of the salicylic acid (SA) pathway [66]. Alterations to sphingolipids, such as the modulation of ceramide production, enable the activation of SA-upregulated *PDLP5* and result in PD closure by increasing PDC deposition as a defense system against powdery mildew infection. However, further study is required to fully understand the mechanism by which sphingolipids control PDC homeostasis. Overall, these insights could be used to develop new hypotheses for studies on the role of plant lipid rafts in PDC turnover and PD regulation.
